# Selected Aspects in the Pathogenesis of Autoimmune Diseases

**DOI:** 10.1155/2015/351732

**Published:** 2015-08-02

**Authors:** György Nagy, Peter C. Huszthy, Even Fossum, Yrjö Konttinen, Britt Nakken, Peter Szodoray

**Affiliations:** ^1^Department of Genetics, Cell and Immunobiology, Semmelweis University, Budapest 1089, Hungary; ^2^Centre for Immune Regulation and Department of Immunology, University of Oslo and Oslo University Hospital, Rikshospitalet, 0372 Oslo, Norway; ^3^K.G. Jebsen Center for Influenza Vaccine Research, Institute of Immunology, University of Oslo and Oslo University Hospital, 0372 Oslo, Norway; ^4^Department of Medicine, Institute of Clinical Medicine, Helsinki University Central Hospital and ORTON Orthopaedic Hospital of the Invalid Foundation, 0280 Helsinki, Finland

## Abstract

Autoimmune processes can be found in physiological circumstances. However, they are quenched with properly functioning regulatory mechanisms and do not evolve into full-blown autoimmune diseases. Once developed, autoimmune diseases are characterized by signature clinical features, accompanied by sustained cellular and/or humoral immunological abnormalities. Genetic, environmental, and hormonal defects, as well as a quantitative and qualitative impairment of immunoregulatory functions, have been shown in parallel to the relative dominance of proinflammatory Th17 cells in many of these diseases. In this review we focus on the derailed balance between regulatory and Th17 cells in the pathogenesis of autoimmune diseases. Additionally, we depict a cytokine imbalance, which gives rise to a biased T-cell homeostasis. The assessment of Th17/Treg-cell ratio and the simultaneous quantitation of cytokines, may give a useful diagnostic tool in autoimmune diseases. We also depict the multifaceted role of dendritic cells, serving as antigen presenting cells, contributing to the development of the pathognomonic cytokine signature and promote cellular and humoral autoimmune responses. Finally we describe the function and role of extracellular vesicles in particular autoimmune diseases. Targeting these key players of disease progression in patients with autoimmune diseases by immunomodulating therapy may be beneficial in future therapeutic strategies.

## 1. Introduction

Autoimmune diseases are typically multietiological entities, where genetic and environmental abnormalities along with derailed immunoregulatory processes contribute to the development of disease. In the healthy immune system, various tolerance mechanisms, such as activation-induced cell death, anergy, or clonal ignorance, play a protective role to prevent the activation of self-reactive lymphocytes [1]. In autoimmune conditions, self-reactive lymphocytes may not be subjected to the aforementioned tolerance mechanisms raising the possibility of the survival and activation of autoreactive T and B cells upon autoantigen encounter [2–4]. However, there is a fine line between autoimmune processes, which also appear in healthy individuals and manifested autoimmune diseases. In autoimmune diseases, one or several tolerance mechanisms permanently fail due to the constellation of various environmental factors, specific HLA- and non-HLA genes and/or derailed immunoregulatory processes, leading to the persistence of self-reactive T- and B-cell clones and ultimately organ damage [4, 5]. Immunoregulatory abnormalities and/or the imbalance of immunoregulatory and inflammatory processes could lead to the progression towards autoimmune diseases. Besides faulty tolerance mechanisms, several other factors, such as imbalance of the pro- and anti-inflammatory cytokines, extracellular vesicles, abnormal autoantigen scavenging machinery, and antigen presentation, can contribute to the development and perpetuation of autoimmune processes and eventually to the progress towards autoimmune diseases. Herein we aim to address some selected pathogenetic points in the development of autoimmune diseases.

## 2. Animal Models of Autoimmunity

Acquired immunity has evolved with an intricate control system to balance pro- and anti-inflammatory responses. Autoimmunity or immunity toward “self” is a pathological process that involves autoreactive B cells and corresponding autoantigen-specific T cells, imbalances in cytokine levels, and a shifted leukocyte polarization profile. In most of these diseases, a proinflammatory environment dominates, with a Th1 (type 1 insulin-dependent diabetes mellitus, Hashimoto's thyroiditis), Th17 (multiple sclerosis), or combined Th1/Th17 (Sjögren's syndrome) signature. Animal models of autoimmunity have been important research tools for many years now, aiding to pinpoint various components of the pathogenesis of human autoimmune diseases. Today, more than 80 types of autoimmune pathologies are recognized, most with distinct clinical profiles. Animal models have been developed for all the major disease entities, for example, type 1 diabetes mellitus (T1D), rheumatoid arthritis (RA), multiple sclerosis (MS), Sjögren's syndrome (SS), and systemic lupus erythematosus (SLE). Based on the etiological background and induction of symptoms these animal models can be divided into three broad categories: spontaneous, induced, and genetically engineered. The strengths and weaknesses of each are briefly discussed below.

### 2.1. Spontaneous Models of Autoimmunity

Susceptible rodent strains spontaneously develop autoimmunity. Well-known examples include the NOD mouse that develops T1D and inbred mice (MRL/*lpr*, NZB/KN, and Biozzi) that develop arthritis. NOD mice have been extremely useful in delineating the basic principles of T1D [1]. As in humans, so in both NOD mice and susceptible BB rats, T1D is dependent on a collection of genetic traits rather than a single gene [6]. The dependence of T1D on autoreactive T cells and the resolution of disease through T-cell modulation have been important proofs of principles observed in NOD mice. In NOD mice, the sequential development of human T1D is well recapitulated, starting with peri-insulitis (leukocytic infiltration into periductal areas, 3–6 weeks) followed by intrainsulitis (islets are invaded by inflammatory cells recognizing *β*-cell autoantigens) and diabetes (T cell-mediated destruction of islets, 10–30 weeks).

A recent spontaneous model for MS is the relapsing-remitting mouse (RR mouse). RR mice harbor increased frequencies of myelin-specific CD4 T cells and are on a SJL/J background [7]. This model is unique, as it resembles human MS in the dependence on autoreactive B cells and the production of autoantibodies and develops without artificial induction [8]. A proof of principle for the contribution of gut microbiota to MS development by T-cell activation has been shown using this model system [9]. Along these lines the contribution of viruses or bacteria as triggers for autoimmune diseases is an important field of study that would benefit from animal models.

### 2.2. Induced Models

Systemic autoimmunity may be induced in rodents after injection of the lipid pristane (tetramethylpentadecane; leading to SLE and RA), mercury, or foreign grafts (leading to graft versus host disease, GVHD). Animal models of organ-specific autoimmunity are established by administering a tissue-derived self-antigen (or a peptide thereof) together with a strong adjuvant. Well-known models are collagen induced arthritis (CIA) for RA and experimental allergic encephalomyelitis (EAE) for MS. For CIA, intradermal injection of collagen type II together with adjuvant activates autoreactive B and T cells. In susceptible rat strains, such as DA, Freund's adjuvant alone induces arthritis (AA). CIA successfully recapitulates human RA histopathology, with synovial joint inflammation and bone erosion. Regarding the disease course, pristane-induced arthritis more closely resembles chronic, relapsing-remitting human RA, whereas AA usually shows rapid remission. To induce EAE, CNS antigens in adjuvant are used for immunization or myelin-specific Th1 cells are transferred to susceptible strains, such as Lewis rats. In contrast to human MS, which is relapsing-remitting or develops progressively, EAE induced after T-cell transfer to rats has a monophasic disease course [10]. Although proinflammatory lymphocytic infiltration is achieved, the massive demyelination seen in MS is not consistently recapitulated [11]. A major drawback of inducible models is that they are not suitable to elucidate the etiology of the disease and typically fail to recapitulate the full scale of pathological traits associated with human autoimmunity. The disease course in humans with a given diagnosis is often diversified, whereas that in the mouse is highly predictable. Within inbred rodent strains, genetic homogeneity, a homogenous environmental milieu, and the evolutionary distance from humans certainly contribute to this effect.

### 2.3. Genetically Engineered Models

Throughout the 1980s, with the aid of more advanced techniques in cell and molecular biology such as gene cloning and mouse embryonic stem cell manipulation technology, more representative rodent models have been generated. These technologies enabled scientists to generate novel mouse strains with defined genetic changes suspected to contribute to autoimmunity (e.g., expression of defined MHC alleles or selected cytokines). Furthermore, these technologies allowed researchers to clone and sequence T-cell lines specific for autoantigens. The T-cell lines were instrumental for transfer experiments and for engineering T-cell receptor knock-in mice [12]. Transgenic mice that express defined human or mouse MHC class II alleles have been successful in recapitulating a whole range of autoimmune pathologies, most notably multiple sclerosis, chronic inflammatory bowel disease, and myasthenia gravis [13]. Mice engineered to express full length human TNF-*α* spontaneously develop chronic inflammatory polyarthritis [14]. Proof of principle for TNF-*α* blockade in treating RA has been obtained in this model, an early success story for translational research. Transgenic expression of the human T-lymphotropic virus-1 genome leads to the development of arthritis in mice and this model suggested the role of this virus in the development of human RA [15, 16]. A major advantage of such genetically engineered models is that the induced changes (genes) can be precisely defined and experimentally controlled through comparisons with the parental background strain. Furthermore, they allow for spatial and temporal control of gene expression, through tissue specific or inducible promoters. In addition, expression of a fluorescent or luminescent reporter facilitates* in vivo* imaging approaches.

### 2.4. General Considerations

Ideally, an animal model should reflect the whole range of features associated with human pathology, not only isolated traits thereof. If it is a genetically targeted model, it should rely on homologues of genes/pathways known to be responsible for autoimmunity in humans. Finally, it is desirable that the disease develops spontaneously, so that the etiology of the given syndrome may be investigated. Although none of the animal models have all these features, they have, in concert, been invaluable tools that have shed light on basic disease mechanisms. This has been important, since in many human autoimmune diseases, progression is typically correlated only to serum markers with the pathological tissue being inaccessible (such as T1D or MS) or limited (RA or SS). With the application of more refined imaging techniques, such as PET/CT and intravital microscopy, animal models will continue to provide useful mechanistic insights into metabolic parameters, disease course, and pathways of leukocyte migration. We argue that for any given autoimmune disease, scientific evidence collected from several animal models, inducible, genetically modified, and spontaneous, correlated with clinical parameters and characterization of biopsy/autopsy tissue will be needed to extract more biological information with relevance to human pathology.

## 3. The Role of Regulatory T Cells in Autoimmune Diseases

The fine balance between pro- and anti-inflammatory processes within the immune system is crucial to maintain the antigenic integrity of the individual, while at the same time must effectively eliminate pathogens. A spectrum of characteristic immunocompetent cell populations exists, which have the capability to suppress immune and autoimmune processes. These suppressor activities can be achieved by either cell-cell contact or via anti-inflammatory soluble mediators, for example, anti-inflammatory cytokines. A key member of the family of immunoregulatory cells is denoted as regulatory T cells (Tregs). Tregs derive either from the thymus [CD4^+^CD25^bright^FoxP3^+^ natural Tregs (nTregs)] or the peripheral blood [IL-10, or TGF-*β* producing Type-1 regulatory T cells (Tr1)] [17]. Under pathological circumstances, when quantitative and/or qualitative changes in regulatory T cells and proinflammatory immune responses are evoked, on a susceptible genetic background, autoimmune processes can occur, and eventually various autoimmune diseases can develop. In line with this hypothesis, previous studies on autoimmune disease depicted that the selective decrease in the number of Tregs or, alternatively, a diminished suppressor function of Tregs is characteristic to these diseases (e.g., SLE, Sjögren's syndrome, RA, and MCTD) [18–22]. These data indicate that in patients with established autoimmune diseases a sustained impairment of the regulatory T-cell pool exists.

In order to assess whether regulatory T-cell abnormalities could contribute to the development of autoimmune diseases, it is valuable to assess Tregs in medical conditions, predating the onset of full-blown diseases. In the forerunner medical condition for systemic autoimmune diseases, denoted as undifferentiated connective tissue disease (MCTD), the assessment of Treg cells showed that the percentage and absolute number of natural Tregs (CD4^+^CD25^bright^FoxP3^+^) were diminished in UCTD patients compared with healthy subjects, while the number of inducible Tregs (CD4^+^IL-10^+^) was increased [23]. This progressive divergent shift in natural and induced Tregs clearly predicted the transition from the UCTD, introductory phase to a well-established systemic autoimmune disease [23]. In active systemic autoimmune diseases, the frequency of natural Tregs was found to be decreased compared to healthy individuals or compared to patients with inactive disease [18, 20, 24]. The other major regulatory T-cell subset denoted as IL-10 producing Type-1 regulatory T cells (CD4^+^IL-10^+^, Tr1) has been investigated and found to have an important role in the development of various autoimmune diseases [25]. IL-10, also known as human cytokine synthesis inhibitory factor (CSIF), is a multifunctional cytokine that can suppress the IFN-*γ* production of Th1 cells as well as having other important regulatory functions in differentiation of various T-cell subsets, B cells, or NK cells [26, 27]. Previously we have shown a significant increase in the number of IL-10 producing Tr1 cells in UCTD and we found further increase in patients who progressed into definitive systemic autoimmune diseases. We believe that this phenomenon represents a compensatory mechanism in order to downmodulate the effects of the observed IFN-*γ* overproduction [23].

## 4. The Role of Th17 Cells and the Th17/Treg Ratio in Autoimmune Diseases

Upon particular, proinflammatory conditions, T cells have the ability to differentiate into IL-17-producing T helper cells, denoted as Th17 cells, and this differentiation is independent of Th1 or Th2 cell development [28, 29]. Th17 polarization in humans requires IL-1*β*, IL-6, IL-21, and IL-23, which induce STAT3. While Th17 cells are important in host defense mechanisms against pathogens, the persistent secretion of IL-17 promotes chronic inflammation and is involved in the pathogenesis of inflammatory and autoimmune diseases. Th17 cells recruit neutrophils and macrophages to the site of inflammation; therefore, they are crucial in the initiation of inflammation [29]. As we described, increased levels of Th17 cells and secreted IL-17 have been associated with numerous inflammatory conditions and autoimmune diseases. High levels of IL-17 have been described in the sera, synovial fluids, and synovial biopsies of most RA patients, while osteoarthrosis showed no increased levels of this cytokine [30, 31]. In other systemic autoimmune diseases, IL-17 and Th17 cells may play a role in the pathogenesis, indicated by studies on SLE, or patients with lupus nephritis [32, 33]. In Sjögren's syndrome, an increase in IL-17 has been found in both the serum and the affected salivary glands, indicating that the cytokine may play a part in both development of the glandular and systemic manifestations of the disease [34, 35].

As mentioned earlier, the imbalance of pro- and anti-inflammatory mechanisms, indicated by, for example, Th17 and Treg numbers or function, may initiate and perpetuate autoimmune diseases. Tregs develop in the thymus and participate in the maintenance of peripheral tolerance; however, circulating and local skewed cytokine milieu alters the suppressive function of these cells [36]. Focally, in affected organs of patients with autoimmune diseases increased IL-6 and TGF-*β* expression has been described, which favors the development of Th17 cells. In addition, increased concentrations of TNF-*α*, which is pathognomonic to many autoimmune diseases, downmodulate the function of Tregs, further contributing to the disequilibrium between the pro- and anti-inflammatory processes [36].

A clear shift exists in the cytokine homeostasis in a broad spectrum of autoimmune conditions, fueling the predominance of proinflammatory cells versus Tregs [37–46].

## 5. Cytokine Imbalance, Regulatory/Effector Cells in Various Well-Defined Systemic Autoimmune Diseases

### 5.1. Sjögren's Syndrome (SS)

Sjögren's syndrome (SS) is a chronic, slowly progressive, systemic autoimmune disease that predominantly affects middle-aged women [47]. SS is characterized by mononuclear infiltration and destruction of the exocrine glands, resulting in dry mouth, keratoconjunctivitis sicca, and the presence of other exocrinopathic symptoms [47]. In the pathogenesis, different subsets of T and B lymphocytes and monocytes play a pivotal role. Increased cell activation, disproportional programmed cell death, and faulty autoantigen scavenging are important in the pathogenesis and processes that are partly driven by a skewed cytokine milieu [48, 49]. A group of peripheral cytokines, chemokines, and growth factors have been implicated in the pathogenesis of SS, contributing to the perpetuation of the cellular and humoral autoimmune processes [49–56]. Skewed T-cell subsets and cytokine imbalance seem to play important roles in an orchestrated proinflammatory cascade in SS. Among circulating cytokines, high IFN-*γ*, along with reduced levels of IL-10, has been described in SS [53]. Moreover, circulating cytokines have the ability to distinguish SS patients with ectopic salivary gland germinal centers, a possible forerunner of lymphoma development in the disease [35, 46, 55]. In patients with SS the following mediators seem to be increased compared to healthy subjects: IL-1*β*, IL-2, IL-6, IL-15, IFN-*γ*, and CCL4 (MIP-1*β*) [35]. pSS patients with ectopic germinal center formation were distinguished from healthy individuals by higher levels of IL-4, IL-10, GM-CSF, IFN-*α*, CCL3 (MIP-1*α*), CCL11 (eotaxin), and B-cell activating factor (BAFF/BLyS), while germinal center positive and negative pSS patients differed in CCL2 (MCP-1) expression. The biomarkers having the strongest discriminatory power amongst SS patients with or without ectopic salivary gland germinal centers were CCL11 (eotaxin) and IFN-*γ*, as well as BAFF/BLyS [35]. Taken together, these findings suggest that a group of mediators (e.g., cytokines and chemokines) has the ability to steer a proinflammatory milieu in these patients, presumably contributing to a derailed Th17/Treg balance.

### 5.2. Systemic Sclerosis

Systemic sclerosis (SSc) is a systemic disease of autoimmune pathogenesis, characterized by excessive extracellular matrix deposition and damage of the small blood vessels, leading to inflammatory processes, dominantly in the skin and visceral organs, such as the heart, lungs, or kidneys [57, 58]. Immunoregulatory abnormalities have been depicted in the pathogenesis of SSc [59–62]. Disorders of the immune system lead to chronic inflammatory processes, abnormal T-cell activation, B-cell abnormalities, abundant production of proinflammatory cytokines (e.g., IL-4), and the production of characteristic autoantibodies. Tregs with impaired function have been shown to play a role in the initiation and perpetuation of the disease [61, 62], while increased levels of circulating Th17 cell have been described, along with elevated IL-17 serum concentrations [63, 64]. An altered balance of the Th1 and Th2 cytokines may also be responsible for the development of fibrosis [65]. Patients with SSc had higher percentages of activated T cells, in addition to a population shift between the effector and regulatory T cells. Increased Th17 cell percentages, together with decreased levels of Th1, as well as regulatory T-cell subsets were characteristic of these patients [66]. Interestingly, the functional assessment of Tregs identified that the suppressor activity of Tregs was clearly decreased in SSc, compared to that of healthy individuals. Our data suggest that the increased Th17/Treg ratio and the altered regulatory function of Treg cells play an important role in the development and progression of SSc [40, 66, 67].

### 5.3. Mixed Connective Tissue Disease

In mixed connective tissue disease (MCTD) the most frequently observed symptoms are arthritis, Raynaud's phenomenon, myositis, esophageal dysmotility, and acrosclerosis along with the presence of autoantibodies reactive with U1 small nuclear RNP (U1RNP) autoantigens [68–76]. Previously we assessed serum cytokines and intracellular cytokine production of CD4^+^ and CD8^+^ T cells in patients with MCTD [76]. Serum concentrations of both type 1 and type 2 cytokines were significantly higher in patients with MCTD than in healthy controls. The percentage of IL-10-producing CD4^+^ and CD8^+^ T cells was higher in patients than in controls. In addition, CD4^+^ and CD8^+^ T cells from patients with active MCTD produced significantly more IL-10 than cells in patients with inactive disease or in healthy individuals [76]. MCTD is characterized by a wide spectrum of T-cell abnormalities, which becomes explicit in the active phase of the disease. Concerning the role of immunoregulatory abnormalities in the pathogenesis, we assessed Tregs in patients with MCTD. The percentage and the absolute number of natural Treg cells were lower in patients than in healthy controls which further decreased in patients with active disease. Interestingly, we saw an increase in inducible, IL-10 secreting CD4^+^IL-10^+^ Tr1 cells in patients with MCTD. The Tr1 cells ratio further increased in patients with active disease. As we indicated in patients with UCTD, we believe that elevated Tr1 cell percentages could be a compensatory mechanism aiming to restore the balance between type 1 and type 2 cytokines in MCTD [18]. In subsets of MCTD patients, serum levels of IFN-*γ* and TNF-*α* were increased along with reduced number of Tregs. The decreased levels of regulatory T cells, along with the increased expression of proinflammatory cytokines, may play a role in the pathogenesis of immune mediated inner ear disorders in MCTD [77].

Serum and intracellular cytokine assessment, reflecting immune-regulatory abnormalities, are valuable biomarkers to assess disease activity in MCTD and are also capable of subcategorizing these patients [78].

### 5.4. Systemic Lupus Erythematosus (SLE)

SLE is a heterogeneous, systemic autoimmune disease with various organ involvements, encompassing mild to moderate forms and also severe, progressive variants [79–81]. A great variety of cytokines have also been implicated in the pathogenesis of SLE, amongst others, BAFF/BLyS, TNF-*α*, IFN-*α*, IFN-*γ*, IL-12, IL-23, IL-18, IL-6, IL-10, and IL-17 [82, 83].

Pinpointing the key role of the interferon signature in SLE, interferon regulatory factor-5 (*IRF5*) has been linked to the increased production of interferon- (IFN-) *α*, and* STAT4* to the increased sensitivity to IFN-*α* [84–86]. Patients with disease flare had significant alterations in a wide variety of soluble mediators at baseline with significantly higher levels of proinflammatory mediators, including Th1-, Th2-, and Th17-type cytokines, already several weeks before the appearance of clinical flare compared to clinically stable patients [87]. Regulatory cytokines, including IL-10 and TGF-*β*, were higher in nonflare SLE patients [87]. Treg-cell number and function have been shown to be impaired in SLE [88–90] along with increases in Th17 cells and serum IL-17 concentrations, in particular in patients with disease flare [91–93]. The Th17 and Treg ratio indicates that SLE is associated with a reduction in the levels and function of immunosuppressive Treg cells together with an increase in the proinflammatory Th17 cells [94].

As we have illustrated, in all these various patient groups with autoimmune conditions, we could identify a circulating cytokine imbalance, a proinflammatory milieu, and the development of Th17 cells along with the reduction in numbers/function of Tregs. The simultaneous, opposing effect of Th17 cells and Tregs has a strong impact on immune homeostasis, deciding and controlling the development of autoimmunity in these patients.

## 6. Dendritic Cells and Autoimmunity

Dendritic cells (DC) are generally divided into two main classes, the plasmacytoid DCs (pDCs) that react to viral infections by secreting large amounts of type I interferons and conventional or classical DCs (cDCs) that are important for initiating immune responses through their ability to take up and present antigen to antigen-specific T cells. For efficient priming of T cells and induction of effector cells, the cDCs have to become activated via signals delivered through pathogen recognition receptors such as toll-like receptors (TLRs). Activated cDCs express large amounts of peptide-MHC complexes on their surface and in addition upregulate costimulator molecules such as CD80 and CD86 for efficient priming of antigen specific T cells. Bacterial and viral infections produce a large amount of TLR ligands, such as LPS (TLR2), CpG (TLR9), or dsRNA (TLR3), resulting in activation of cDCs and efficient induction of effector T cells. In the absence of activation, however, it is believed that cDCs instead promote tolerance, through either induction of Treg or T-cell unresponsiveness [95–97]. cDCs therefore play a central role in determining the outcome of the immune response and whether the resulting T-cell responses are immunogenic or tolerogenic.

### 6.1. Subsets of DCs

Since their initial discovery by Steinman in the early 70s [98], a growing body of information now results in cDCs being divided into subsets based on tissue distribution, surface receptor expression, and functional profiling. In general, cDCs can be divided into lymphoid tissue DC (LT-DCs), in addition to nonlymphoid tissue DC (NLT-DC), or migratory DCs, which source antigen in the periphery and subsequently migrate to draining LN for T-cell priming. LT-DCs were initially divided into subsets based on expression of CD8*α* [99] and CD4 [100]. These makers are, however, not optimal when classifying NLT DCs in peripheral organ, with expression of the integrins CD103 and CD11b being more efficient at separating NLT DC populations [101, 102]. While a number of different surface marks have been evaluated, it now seems that the chemokine receptor Xcr1 is one of the more selective makers for identifying LT CD8*α* ^+^ DC and the related NLT CD103^+^ DC population [103, 104] and that the integrin Sirpa (CD172a) can be used as a marker for CD4^+^/CD11b+ DC [105]. The expression of the markers Xcr1 and Sirpa has the added advantage of being conserved on homologous DC subsets in other species such as man [106, 107], macaques [108], and sheep [109], making for a more unified DC nomenclature across the species.

Both Xcr1^+^ and Sirpa^+^ DCs efficiently present antigens to CD4^+^ T cells [110]. However, functional analysis has indicated that only the Xcr1^+^ DC excel at cross-presenting antigen to CD8^+^ T cell and have subsequently been referred to as cross-presenting DCs [110, 111]. Similar observations have also been made in peripheral tissue with NLT Xcr1^+^ DC (defined as CD103^+^ DC) being better at presenting antigen to CD8^+^ T cells [112]. Whether the same functional difference remains conserved on DC subsets in humans remains a matter of debate, with some studies demonstrating that Xcr1^+^ DC are better at cross-presenting antigens [107], while others have shown similar cross-presentation abilities in Xcr1^+^ and Sirpa^+^ DCs [113].

Dendritic cells have been suggested to be involved in the onset of several different autoimmune diseases [114]. However, the mechanisms by which DC play a role in autoimmunity may vary between diseases. As antigen presenting cells, DC may directly prime autoimmune T cells, but as indicated earlier they may also induce Tregs that inhibit autoimmune disease. In addition, DCs may secrete proinflammatory cytokines upon activation which may be directly involved in disease progression. Here we depict a few examples on the possible involvement of DCs, the progression of type 1 diabetes (T1D), and systemic lupus erythematosus (SLE).

### 6.2. Type 1 Diabetes (T1D)

Type 1 diabetes is thought to be initiated by the presentation of diabetogenic antigens in the draining pancreatic lymph node (pLN), with subsequent induction of autoreactive T cells which destroy the pancreatic *β*-islet cells leading to loss of insulin production and diabetes. Conditional depletion of DCs has been reported to prevent disease onset in diabetes prone NOD mice, suggesting that DC may be involved in the initial priming of autoreactive T cells [115]. Interestingly, a recent study observed that Batf3 deficient NOD mice did not develop diabetes [116]. Mice deficient in the transcription factor Batf3 lack the Xcr1^+^ LT and NLT DC populations [117], suggesting involvement of this DC subset in the onset of disease. Ferris and colleagues observed that islets of Langerhans contained a minor population of Xcr1^+^ NLT DC, in addition to a major population of macrophages. Deletion of the Xcr1^+^ NLT DC in the NOD.Batf3^−/−^ mice resulted in absence of presentation of MHC-I epitopes in the pLN and no incidence of diabetes. These observations are in accordance with previous studies suggesting that cross priming is required in the pathogenesis of diabetes [118] and the functional specialization of Xcr1^+^ NLT DC in terms of cross presenting antigen to CD8^+^ T cells.

### 6.3. Systemic Lupus Erythematosus (SLE)

While lupus is generally thought to be an autoantibody driven disease, studies have shown that certain MHC haplotypes display a strong association with disease onset suggesting that T cells may also play a role in the pathogenesis [119]. In lupus prone MRL.Fas^lpr^ mice, depletion of DCs resulted in less severe disease development with lower glomerular and interstitial renal disease scores [120]. Interestingly, the authors observed that DC depletion did not significantly influence T-cell activation but that T-cell expansion was severely reduced. Consequently, the immunogenic role of the DCs seems to outweigh the tolerogenic function of DC in this model of autoimmunity. It should be noted that depletion of DCs in this model only ameliorated the disease, suggesting that other cells subsets may be involved in the initiation events. Maybe more surprising was the observation that DC depleted mice displayed reduced numbers of plasmablasts in spleen and inhibited class switching, suggesting that the DCs may be involved in this process. Previous studies have indicated that DC may play a role in plasmablast differentiation in SLE [121], which corresponds well with the observations by Teichmann et al. While DCs in autoimmune disease progression tend to primarily function as antigen presenting cells (APCs) and activators of T cells, it is clear that their stimulatory effect on B cells may also affect disease progression.

Type I interferons play a major role in disease progression in SLE, and patients with high levels of type I interferon in serum have more severe disease outcome [122]. As highly efficient producers of type I interferons, pDCs have been suggested to be an important source of type I interferon and disease onset in SLE [123]. In human patients, pDCs have been observed to accumulate in the tissue lesions suggesting a possible involvement in lupus [124]. While most observations implicating pDCs in SLE progression have so far been indirect, a recent paper by Sisirak and colleagues showed that impairing pDC function in a murine SLE model nearly abolished disease manifestations such as glomerulonephritis and anti-DNA antibodies [125]. These observations clearly indicate that pDCs are directly involved in SLE pathogenesis and the induction of autoantibodies.

## 7. Extracellular Vesicles in Autoimmune Diseases

Extracellular vesicles (EVs) are heterogeneous, membrane surrounded structures that can be found in body fluids and play a crucial role in the intracellular communication [126, 127]. Based on their morphological parameters and biogenesis EVs can be categorized as exosomes, microvesicles (MVs), and apoptotic bodies. Exosomes are the smallest EVs; their size range is around 50–100 nm, similar to the size of the viruses. Exosomes are released by exocytosis of multivesicular bodies [128], and they may transfer viruses and microRNA (miRNA). Exosome markers include lysosomal-associated membrane protein 1 (LAMP-1), CD63, and CD81. Among others, several tumor cells and immune cells release exosomes constitutively or upon activation.

The size range of microvesicles (MVs) is between 100 and 1000 nm, similar to the size of bacteria and immune complexes. Detergent treatment (0.05% Triton X-100) completely eliminates the MVs; while it does not significantly influence the immune complexes [129], this method can be used to discriminate MVs and immune complexes. MVs are generated by budding of the plasma membrane. Although platelets, endothelial cells, and red blood cells are the primary sources of MVs, many other cells may release these structures as well. MVs have a central role in the fetomaternal communication and in the pathogenesis of several autoimmune diseases, such as RA and SLE. Phosphatidylserine externalization is a general feature of the MVs. Apoptotic bodies are the largest EVs; their size range is between 1 and 5 *μ*m (the size range of platelets); they are secreted during apoptosis. Similarly to MVs apoptotic bodies also express phosphatidylserine in the outer part of their membrane and they generally contain DNA. Apoptotic bodies have a central role in the transfer of DNA and oncogenes between various cells. Flow-cytometry, western blotting, ELISA, electron microscopy, and mass spectrometry are the most frequently used methods in the EV research. Despite the widespread research of EVs there are still many technical pitfalls and the researchers have to be especially careful when interpreting their results.

There are increasing data on the potential pathogenetic role of MVs in RA and in SLE. Increased number of platelet derived MVs (PMVs) in the serum and synovial fluid samples of patients with RA were reported [130, 131]. The number of PMVs was increased in the plasma samples of patients with RA compared to the healthy donors', and the number of PMVs correlated with the disease activity [132]. The PMVs have procoagulant activity; therefore PMVs may contribute to the increased cardiovascular risk in RA. It was recently published that C-type lectin-like receptor 2 (CLEC-2) is expressed on the MVs of patients with RA [133], and the authors suggest using this marker to measure PMVs. In general, most studies on MVs in RA investigated morphological features and there are only relatively little data available about their biological effects. Fibroblast-like synoviocytes of patients with RA produced B-cell-activating factor upon MP treatment, suggesting that these structures may contribute to the activation of the adaptive immune response in RA [134].

The number of MPs is increased in SLE and the protein composition of them is different from the healthy donors' [135]. The number of endothelial MVs was measured in a recently published prospective study by flow cytometry [136]. The number of EMPs was higher in the samples of patients with SLE than in the samples of healthy donors. Disease activity decreased during the study and in parallel the number of EMPs decreased. Importantly, the clinical disease activity (BILAG-2004 and SLEDAI-2K scores) did not correlate with the EMP numbers. These data suggest that endothelial dysfunction may improve with the appropriate reduction of the inflammation in SLE. Increased number of MVs were also reported in systemic sclerosis [137] and polymyositis [138] and in Sjögren's syndrome [139].

## 8. Conclusions

The intricate interplay of various proinflammatory cytokines and chemokines, orchestrated by key regulators of the immune system (e.g., dendritic cell subsets), can lead to the imbalance between regulatory (e.g., Tregs) and proinflammatory cells (e.g., Th17 cells). This vicious circle can further perpetuate autoimmune processes, which, on a susceptible genetic background, can lead to the development of a full-blown autoimmune disease. Other novel mediators, denoted as extracellular vesicles, seem to have a pivotal role in the pathogenesis, as well. The fine mapping of these mediators does not just help us to understand the pathogenesis of these diseases, but we believe that long, empirical therapies can be replaced by optimized combination therapies through personalized proinflammatory cytokine, extracellular vesicle targeting, or dendritic cell manipulation. We believe that this approach will aid in the diagnosis and therapy design in autoimmune diseases and will provide an advanced disease management in the future.
